# Outpatient care of adults with congenital heart disease in the UK: a qualitative appraisal of the clinician perspective

**DOI:** 10.1136/openhrt-2023-002420

**Published:** 2024-01-29

**Authors:** Isobel Chaudhry, Anisa Ghassani, Jo Wray, Bill Chaudhry, Louise Coats

**Affiliations:** 1 Population Health Sciences Institute, Newcastle University, Newcastle upon Tyne, UK; 2 Heart and Lung Directorate, Great Ormond Street Hospital for Children NHS Foundation Trust, London, UK; 3 Institute of Cardiovascular Science, UCL, London, UK; 4 Biosciences Institute, Newcastle University, Newcastle upon Tyne, UK; 5 Adult Congenital Heart Unit, Freeman Hospital, Newcastle Upon Tyne Hospitals NHS Foundation Trust, Newcastle Upon Tyne, UK

**Keywords:** Heart Defects, Congenital, Quality of Health Care, Health Services

## Abstract

**Objective:**

This study aimed to explore clinicians’ perspectives of ambulatory care in adult congenital heart disease (ACHD).

**Methods:**

Semistructured interviews were carried out remotely (Zoom) with a range of physicians providing ambulatory care to patients with ACHD across the UK. The chronic care model, thrive and candidacy frameworks were used to design prompt guides and subsequently develop themes. A framework approach was used to code and analyse transcripts, which were managed in NVivo.

**Results:**

21 clinicians (43% females, 38% specialists) from 10/12 ACHD networks in the UK participated. Shared themes included the purpose of the clinic appointment, problems in the ‘hub-and-spoke’ care system, role of the general practitioner and ACHD specialist nurse, communication with patients, burden of ambulatory care and patient self-management. Reflecting on these themes, participants identified resources, what care and how and by it is delivered alongside the role of the patient as key areas for future research.

**Conclusions:**

The present structure of ACHD ambulatory care is neither patient-centred nor equitable. The concerned clinicians raise the question whether increasing resource alone without changing structure will lead to better outcomes for patients.

**WHAT IS ALREADY KNOWN ON THIS TOPIC**

- Adults with congenital heart disease need lifelong cardiac surveillance and in many cases support for associated comorbidities. The present clinic structure is characterised by gaps in follow-up, non-attendance and a high burden of care for patient and provider. Preferences of patients regarding their care delivery are presently being collected.

**WHAT THIS STUDY ADDS**

- Understanding attitudes of clinicians caring for those with adult congenital heart disease (ACHD) to the present ambulatory care system are critical before implementing change. Unlike other chronic conditions, we found limited involvement of local physicians and general practitioners in the care of those with ACHD. Although attempts to address this imbalance are mediated by a ‘hub-and-spoke’ model of care, presently holistic care is not achieved as there is adherence to the traditional clinic structure led by the ACHD specialist. Communication between clinicians is further hindered by differing electronic records and modes of communication. There is seen to be inequity in access for different demographic groups and opportunities for self-management are limited.

**HOW THIS STUDY MIGHT AFFECT RESEARCH, PRACTICE OR POLICY**

- Clinicians caring for those with ACHD acknowledge significant concerns with the current model of ambulatory care delivery. Alternative approaches, considering the expressed needs of the patients as well as successful models from other chronic conditions, must be tested in ACHD to enable flexible, patient-centred, evidence-based care delivery that is the aspiration of all stakeholders.

## Introduction

Contemporary epidemiology of adult congenital heart disease (ACHD) confirms a growing, increasingly complex, ageing population with significant care needs.^1^ Many have multiple long-term conditions, due to syndromic CHD and coexisting cardiometabolic and clinical mood disorders.^2^ Almost all with ACHD require ongoing surveillance for complications including valvular dysfunction, arrhythmia and heart failure. The heterogeneity of underlying conditions means relevant prognostic information is limited and clinical guidelines are almost entirely consensus driven.^3^ Delivery of ACHD surveillance via the traditional outpatient clinic model generates a high care burden for both patient and provider, particularly as expertise is limited to a small number of specialist centres. This current model of care delivery is not outcome driven,^4^ although outcomes that matter and are relevant to contemporary ACHD populations are starting to be defined.^5^ This study explores clinicians’ perspectives of ambulatory care in ACHD.

## Methods

### Participants

Physicians caring for patients with ACHD in the UK were approached for inclusion. These comprised specialists working in ACHD surgical centres; cardiologists with varying expertise working in non-surgical ACHD centres; cardiologists working in hospitals with no ACHD service and general practitioners (GP). The study was advertised to cardiologists sequentially via CHD networks and the ACHD consultant group. GPs were approached via the Primary Care Cardiology Society, local primary care networks, Royal College of General Practitioners Clinical Advisory Group and the Academic GPs network (figure 2). We anticipate this strategy would have reached most if not all ACHD cardiologists, non-ACHD cardiologists linked to CHD care networks and GPs with an existing interest in cardiology, network care or academia. A purposive sampling approach was used to incorporate views from clinicians of different gender, ethnicity, practice and location.

### Interviews and transcription

**Figure 1:**
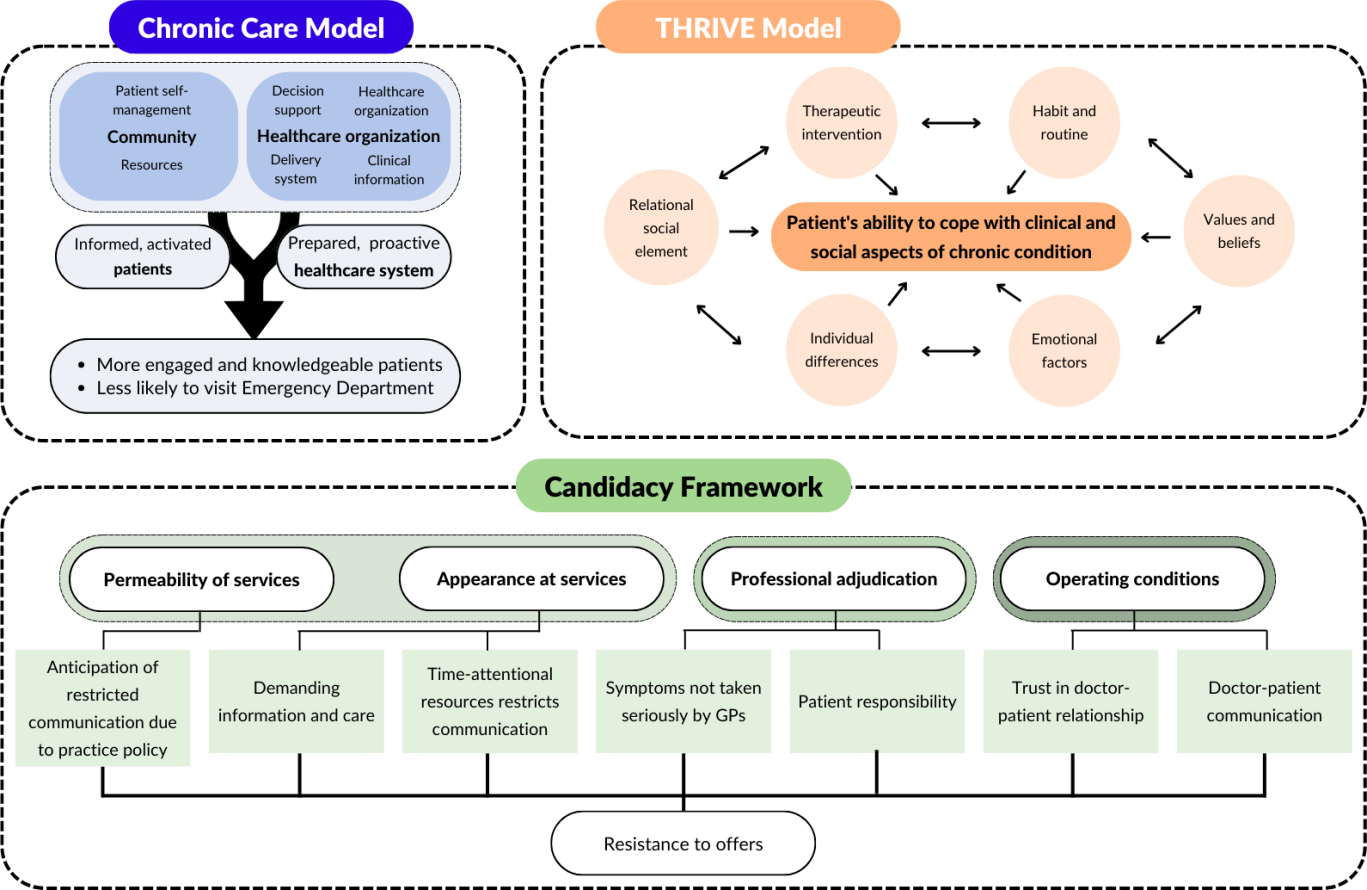
Three theoretical models are used to provide context to the research problem, develop the study design and aid in data interpretation: chronic care model, which empowers the patient and the primary care team and has been shown to produce high-quality care in other chronic disease settings, through a patient-centred, population-based approach,^7^ THRIVE model, which explores six elements that influence how patients cope with clinical and social aspects of chronic disease^8^ and candidacy theory, which considers how people perceive their eligibility to access healthcare, navigate services and assert candidacy, reflecting that difficulties at each stage are typically associated with deprived populations.^9^

Semistructured interviews, lasting 30–60 min, were carried out remotely (Zoom V.5.10.4).^6^ Interviews used prompt guides (online supplemental information) based on three theoretical models relevant to ambulatory care (chronic care model,^7^ THRIVE,^8^ candidacy framework^9^) (figure 1). The software-generated transcript was corrected to match the audio recording and identifiable details were removed. Field notes collected by the interviewer were added as annotations. The number of interviews performed was determined by iterative data review and the study concluded when the research team agreed saturation had been achieved. Participants could review their transcripts to ensure preservation of anonymity without materially changing content.

10.1136/openhrt-2023-002420.supp1 Supplementary data

### Data analysis

Transcripts were analysed and managed in NVivo for Mac (Release V.1.6.1). A framework approach was used, which included data familiarisation, identifying thematic framework, indexing data against framework, charting to summarise indexed data and mapping and interpretation.

The first six interviews were coded by four of the authors (IC, AG, BC and LC). A mixed a priori/emergent method of creating codes was developed by defining parent codes from the theoretical models. New codes were then identified, and initial codes amended through discussion leading to a thematic framework. Interviews were then reviewed to identify where questions were unasked, insufficient answers provided or previously unidentified themes had arisen. Prompt guides were revised to use in subsequent interviews. Remaining interviews were coded by two of the research team (IC and AG). Mapping and interpretation were supported by the ‘One Sheet of Paper’ method to visually identify links and contradictions between themes and subthemes.^10^

## Results

**Figure 2:**
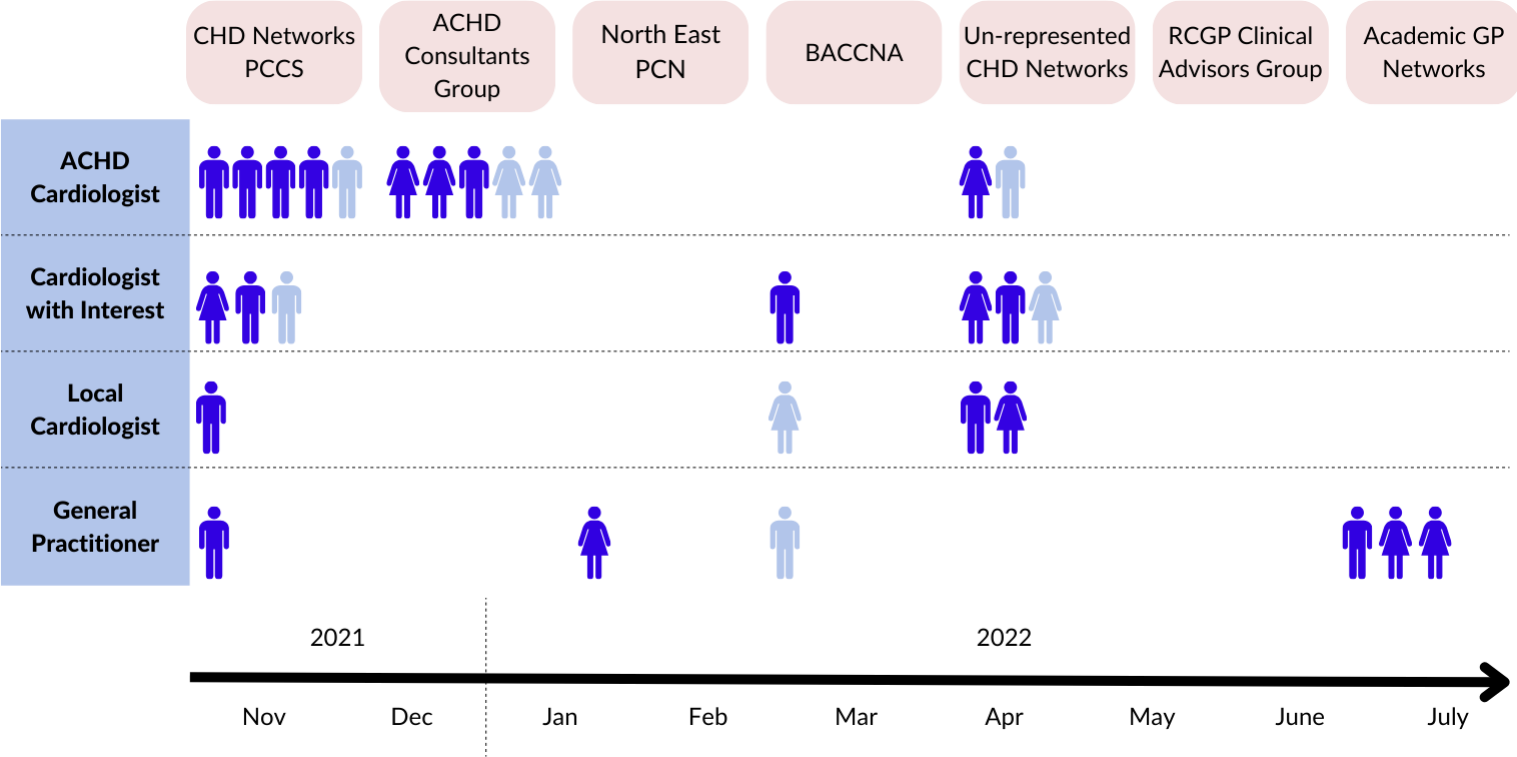
Timeline of subject recruitment. Pink boxes indicate organisations approached to disseminate the study among their contacts. Dark blue symbols indicate participants. Pale blue symbols indicate individuals who expressed interest but did not participate (five unable to find time, two did not respond to follow up emails, one excluded a purposive sampling). ACHD, adult CHD; BACCNA, British Adult Congenital Cardiac Nurses’ Association; CHD, congenital heart disease; PCCS, Primary Care Cardiology Society; PCN, primary care network; RCGP, Royal College of General Practitioners.

Purposive sampling ensured broad representation, with participants recruited from 10 of 12 UK CHD operational delivery networks (table 1, figure 2). Interviews led to identification of several shared themes described from different viewpoints.

**Table 1:** Participant characteristics

Participant characteristics	N (%)
Male	12 (57)
Female	9 (43)
General practitioner	5 (24)
Non-ACHD cardiologist	3 (14)
Cardiologist with an interest in ACHD	5 (24)
ACHD specialist	8 (38)
White British	16 (76)
Other white	3 (14)
Chinese or other Asian groups	2 (10)
Northern	8 (38)
Midlands	2 (10)
Southern	5 (24)
London	3 (14)
Scotland	2 (10)
Wales	1 (4)

ACHD, adult congenital heart disease.

### Purpose of the clinic appointment

Clinic provides surveillance for late complications of CHD to facilitate timely intervention: ‘*Most of it is seeing patients who are at the well-er end of the spectrum, just monitoring their disease and making sure they're okay and making sure nothing bad has happened to them.’* [CI3] However, it also fulfils other purposes: ‘*We might talk about things like risk taking behavior, healthy lifestyle, dental care, reducing the risk of endocarditis, why all that stuff’s important. We might talk about jobs, might talk about family planning, contraception…’* [AS2] Clinicians describe a broader pastoral role provided by a long-standing relationship: ‘*the other part I think is the reassurance that they're actually under a service and under a team…so they have the opportunities, again, to come back and ask questions.’* [AS5] A key need was psychology support, but this was rarely funded: ‘*as a clinician you can see the value of it, but the commissioner side doesn’t see the value of it and so psychological service provision is very limited. I think you've got to be pretty damn far down the line before you'll get any help.’* [CI3]

### Problems in the ‘hub-and-spoke’ care system

Interviewees described how ambulatory care is currently organised by a few UK tertiary surgical hospitals using a ‘hub-and-spoke’ model.^11^ Care may be delivered by specialists travelling to run clinics, with or without participating local cardiologists, or by local cardiologists interested in ACHD working semi-independently. This can impact on the specialist centre. ‘*I go to [LOCAL CENTRE], it’s a 5-hour train ride going there and coming back…I have to cancel my Friday morning clinic to go there and it is for a good reason… but it means that it disrupts the work I do at my centre.’* [AS1] Many areas are poorly served ‘*We have two a year here… and we have on our database about 650 patients. Two a year…so that’s 32 patients a year that can be seen in outreach, and that’s not many.’* [CI4] Local provision was typically determined by the specialist centre, based on disease complexity, resource availability and confidence of local cardiologists. ‘*I wouldn't want to have a complex echo done in a centre where there is no complex echo technician.’* [AS 1] Clinical decision-making was perceived as complicated, exemplified in the ‘hub’ where there is a multidisciplinary team (MDT) approach: ‘*you’ve got your paediatric and adult and often obstetric cardiology as well and dietitians, psychologists, and nurses… because it’s not just you as the cardiologist who makes the decision, it’s a team approach’* [CI2]. This affects the ability of local cardiologists to provide active patient management: ‘*people tend to say, ‘I’ve got no idea what’s going on’, and just refer to a specialist quite quickly.’* [AS2]. Local clinicians often then fulfil an organisational role. ‘*We don't have anybody that would routinely see the ACHD, if there are things that need tidying up in between times … I’ll take that on, but I’m mainly doing administrative work, or kind of acting as the registrar’* [LC3] and in some settings, ‘spoke’ services have discontinued. ‘*Our [LOCAL CENTRE] clinic was shut down when we felt we could not provide the care that we were happy with for our patients. It was a matter of engagement, there was no engagement by the local cardiologists’* [AS1].

### Role of the GP and ACHD specialist nurse

Importantly, GPs, who typically have a well-defined role in co-ordinating care for chronic conditions, often did not feel able to participate ‘*I think they're complex patients, and even for me, I’m a GP specialist in cardiology, it’s not my specialist area.’* [GP2] Moreover, ACHD specialists did not expect them to actively contribute: ‘*the GP is informed, but not expected to be proactive.’* [AS7] The clinic letter may also not be the most effective way to communicate ‘*I’m sure you know that most of the letters that come into practices aren't even seen by GPs anymore, they're actually done by coding clerks’* [GP1].

ACHD specialist nurses bridge the gap between different clinicians and the patient ‘*[Specialist nurses] are generally very helpful… if you think a patient needs to see a consultant, then I think speaking to them and them advocating that for you seems to work better than anything else.’* [GP4] ‘*Sometimes, the communication will go directly to a specialist nurse, and then she'll speak to me, and we'll organize what’s needed.’* [CI4]

### Communication with patients

*‘The things that they [the patients] most appreciated, was some kind of personal touch and some kind of, you know, human communication and continuity.’* [AS8] Continuity in relationships, adequate consultation time, language used and accessibility of services were identified as critical to patients engaging with care. Providing these elements was difficult and expected to worsen with population growth. ‘*The times when things went horribly wrong is when there were multiple different people looking after [patients] with limited continuity, limited specific time invested to explain, or to help, or to coordinate, or to simplify processes… and that’s usually a systematic failure, rather than individual or clinical failure.’* [AS8] Interviewees acknowledged personal uncertainties communicating health information to patients. ‘*And that’s the hard thing, it’s very hard to have a crystal ball and say your valve will last you the rest of your life, or your valve will last you twelve months or whatever. And that’s really hard, patients want black and white, but in reality, medicine is pretty grey really.’* [CI 2] Disparities in electronic patient records between organisations were cited as key to delays and communication problems ‘*the electronic patient record systems are all different, and that’s you know that’s where documentation can get lost…’* [AS5] Written communication was not always effective. ‘*I think the majority of our patients, either have not received communication because we still don't have emails for them’* [AS1].

### Burden of ambulatory care

Complexity in ACHD is due to the range of cardiac conditions together with variability in age, location and social setting of the patient. Social support can impact clinic attendance with costs to patients and carers well-documented.^12^ ‘*It’s usually the same people, often deprived, often from low-income families and things’* [CI2]. One-stop clinics tailored to diagnostic subgroups aim to reduce care burden ‘*We tend to day case our Fontans, in the sense of organising repeat investigations in clusters.’* [AS1]. However, such clinics may inadvertently introduce inflexibility: ‘*you know you can't just have your tetralogy clinic and then your arterial switch clinic or your systemic RV clinic because then the patient doesn't have the choice about when they come’* [AS8]. The COVID-19 pandemic led to virtual clinics which decreased care burden. ‘*The vast majority is ‘see you next time’…I mean you need to make it easier for them and yourself and I think video solution has made it easier…’* [AS1]. However, retention of these clinics post-COVID-19 was determined primarily by physician preference. ‘*I enjoy the face-to-face interaction more. I didn’t like the telephone consultations as much, and I haven’t found that it saves much time…’* [AS5]. Quotes illustrating challenges patients and clinicians face engaging in ACHD ambulatory care are shown in table 2.

**Table 2:** Quotes from participants reflecting challenges patients and clinicians face in the present ambulatory care system set in the context of elements of the candidacy framework

Candidacy framework	Quote	Participant
Identification of candidacy	Sometimes, we think that people have a reasonable understanding of their heart condition and then, you know, you might find out later on that they don't. It’s always hard to gauge…	CI 5
Access to service	A lot of the ACHD patients, are often expected to travel long distances … They're often the kind of population that aren't in a position to do that…young people with work commitments, children, they might not have English as their first language, or they may have other comorbidities.	LC 3
Accessibility of services	So, inflow to the clinic is via direct referral from other cardiologists or from transition clinic, or via established follow up and/or transfer from another ACHD unit.	AS 8
Appearance at services	I think for a lot of people it’s, well, managing symptoms and avoiding admission where possible, you know, there’s not many patients that want to go into hospital.	GP 4
Professional responses	I think that face to face interactions are a really important part of care, you know… it’s [video] fine for listening to a talk or a lecture or something like that, but it’s not fine for delivering care to people	CI 3
Sustainability of resources	The problem with ACHD is that the number of patients are growing and the number of specialists is not really growing.…	CI 3
Doctor–patient relationships	…and with ACHD, these patients are very chronic, they’ve had these conditions for years, so these professional relationships are very important.	LC 1

ACHD, adult congenital heart disease; AS, ACHD specialist; CI, cardiologist with interest in ACHD; GP, general practitioner; LC, local cardiologist.

### Patient self-management

Patient participation in managing their conditions is recognised but limited ‘*we would encourage people to look at their own blood pressures and we've handed out a few of the Kardia Alivecor devices, in terms of, you know, recording rhythm issues… maybe we're not doing enough?’* [AS4] Specialists worry handing over more responsibility may compound anxiety: ‘*I worry slightly about some of the rhythm monitors…it’s good for some and not good for others, so it increases their sort of number watching and their anxiety.’* [AS8] Clinicians also described challenges, due to experiential differences, in promoting lifestyle interventions. ‘*So culturally, I have found it much more difficult to persuade lifestyle changes to somebody that I couldn’t speak the same language to and couldn’t really culturally identify with*.’ [AS8]

### Reflections on ambulatory care delivery

The aspiration to deliver patient-centred care in ACHD is evident ‘*…and now with things like zoom and Skype … you know, if me as a DGH cardiologist needs some support from an expert, and the patient isn't able to travel, we dial them in, and we have a, kind of, three way call and consultation.’* [LC3], but at the same time many specialists still provide traditional care: ‘*they get asked in for their observations and then asked in for their ECG and Echo separately… when they've had all these things done, they will sit down and then we will talk and go through the usual history, exam, investigations, plan for follow-up.’* [AS7] Across the country, care is heterogeneous and determined by individual expertise and preference: ‘*mostly, or historically, things have just been determined by the primary cardiologist.’* [AS2] Interviewees reflect on insufficient resources for chronic conditions ‘*the government seems very focused on getting the new patient waits down, which is what we're all spending weekends doing, but the return patients, especially in a subgroup like adult congenital patients who have an ongoing issue, is not prioritised.’* [CI4]. A cardiologist whose practice extended beyond ACHD vocalised challenges that changing care also bring for clinicians ‘*within [cardiomyopathy] we’ve had to move to remote screening, which I felt quite uncomfortable about as a clinician because, you know, at the end of the day, I quite like talking to patients, it’s why I got into medicine, and you just lose that personal touch if you’re just writing to patients.’* [LC2] A critical question was then identified by a further participant: ‘*So, it’s not just whether we're delivering face-to-face or video consultation or telephone and how much remote monitoring we can do, but it’s actually who delivers the care as well.’* [AS5].

## Discussion

ACHD is prevalent (3:1000); however, individual conditions are heterogeneous and often rare (<1:2000).^13^ ACHD care has emerged from paediatric clinic models at a small number of tertiary surgical centres. Latterly, the ‘hub-and-spoke’ model, advocated for rare diseases,^14^ has been implemented but our results suggest it is not working adequately for this population.

**Figure 3:**
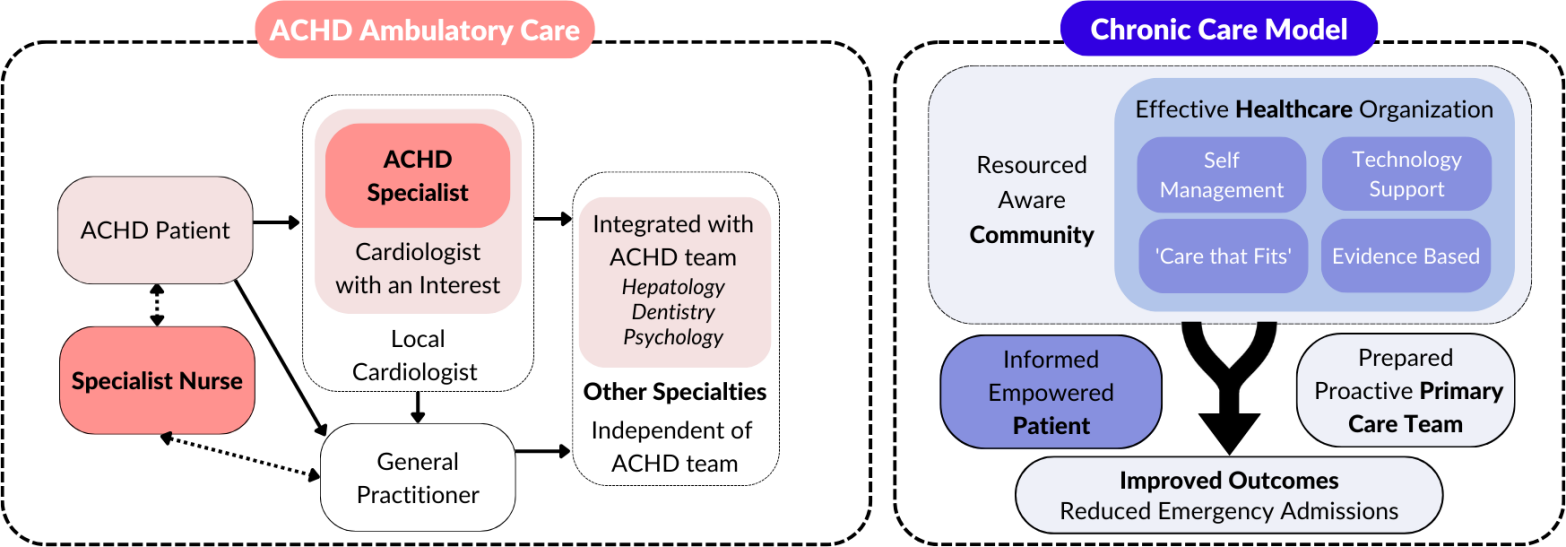
Schematic of ACHD ambulatory care, placed in contrast to the chronic care model. In present, ACHD ambulatory care, key knowledge, responsibilities and decision-making are the remit of the ACHD specialist supported by the specialist nurse (who is not universally accessed by all patients with ACHD) with little or no knowledge, responsibility or decision-making in primary care. In the chronic care model, strong healthcare organisation with key features highlighted in the figure better informs and empowers the patient and elevates the level of knowledge, responsibility and decision making in primary care. ACHD, adult congenital heart disease.

Confidential semistructured interviews provide the ideal methodology with which to secure an in-depth picture of ACHD ambulatory care from the clinician standpoint, as they enable a meticulous understanding of opinions and attitudes across different demographics, expertise and geographies. The structure of ambulatory care, defined by this study, is illustrated in figure 3. Presently, key knowledge, responsibilities and decision-making are the remit of the specialist team, usually located in the tertiary centre. Notably, GPs, who traditionally have primary responsibility for patients with chronic conditions in the NHS, report they are unable to fulfil this role here as decision-making is too complex; local cardiologists similarly report limitations. This dynamic is also observed in other countries and poorer outcomes are observed when non-specialists manage patients with ACHD.^15 16^

Communication appears to be suboptimal and sometimes leads to fragmentation of care and loss of continuity. Clinicians described differing electronic record systems between providers, a wider problem for many specialties.^17^ In streamlining and unifying digital tools, a strategic vision is needed for future-proofing.^18^ Generally, telephone and email communication are preferred over written communication with its known problems.^19^ While many ACHD specialists enjoyed talking to patients, with the importance of lifestyle advice highlighted, it’s not clear if this impacts on the patients’ well-being and whether other groups, such as specialist nurses, psychologists, occupational therapists or physiotherapists may better fill this role. Some clinicians reported difficulty relaying such information due to experiential and cultural differences and felt consultation time was insufficient.

Non-attendance at clinic is concerning as symptoms or new investigation findings usually necessitate intervention; lack of engagement with the present system leads to late presentation and inferior outcomes.^20^ Many reasons are recognised; however, one factor may be that patients are well and for most no actions are taken: they may therefore prioritise competing activities such as work and childcare over the time and travel burden of attending clinic.^12 21^ The lack of defined surveillance pathways in ACHD is an obstacle to remote care provision and reinforces a paternalistic clinician–patient relationship rather than the empowered patient model seen in other chronic conditions.

Alternative ambulatory care models, aligned with the chronic care model,^7^ should be considered. Patient-centred home-based care considers psychosocial and emotional aspects of living with a condition.^22^ It typically involves a GP-led MDT delivering care close to home; one could imagine the GP being supported by allied professionals embedded in the ACHD service accessing the specialist accordingly. Community health workers, linked to primary care or specialised services, can support marginalised and vulnerable individuals to optimise self-management and access services,^23^ worth considering in ACHD perhaps as an extension of the specialist nursing role. Patient initiated follow-up achieves similar health outcomes to standard care across a diverse range of conditions, generally reducing appointments over time, while improving stakeholder satisfaction compared with regular scheduling.^24^ Remote consultation improves access for those living further away, having difficulty with transport or experiencing anxiety attending hospital.^25^ These options and others could surpass the traditional clinic system in parallel with individualised surveillance pathways for anticipated medical complications.

ACHD is felt by many clinicians to be under-resourced with local and national policy prioritising new referrals in acquired heart disease. However, many aspects of the current ACHD ambulatory care organisation were criticised by interviewees, who recognised inequalities between different social groups as a barrier to engagement. Therefore, it is not clear that simply increasing resource in the current system without changing structure would lead to better outcomes or experience for patients. Recent introduction of integrated care boards and CHD operational delivery networks reflect a national move to deliver cross-sector accountable care organisations and integrated care; it is too early to know if this will benefit those with ACHD. New systems must be flexible and adaptable and strategically placed to meet the wide range of medical and non-medical needs of this population. Equally, clear expectations should be set as to what can and should be provided by a public health service and to what degree patient choice can truly be delivered within a highly specialised service.

The study’s sample size is small relative to the total number of clinicians from which they derive, particularly GPs, who were challenging to recruit. It is possible participants self-selected because of specific opinions they wanted to air. However, while interviewees had no difficulty identifying problems with current care, it was interesting to note a general acceptance of the traditional system with few attempts to create change. Qualitative studies are subject to the inherent bias of the authors, we tried to minimise this by drawing the authors from a range of backgrounds (age, gender, ethnicity, geography) and expertise (clinician, psychologist, basic scientist, students) but we acknowledge a different group may have synthesised the data differently and reached different conclusions. This study reports the views of physicians. Future studies must address the opinions of other stakeholder groups including nurse specialists, allied health professionals and the patients themselves, particularly those groups who are often hard to reach, to gain a complete picture of the relevant matters. The conclusions we reached suggest more research is needed to accurately map the population in terms of its needs and accordingly identify first the most effective methods of surveillance, and then how to deliver these according to cost and individual circumstances and preferences.

While no cure exists for the conditions ACHD patients live with, it may be minimal interruptions from the health service but prompt purposeful action when required, is the aspiration of many. Robust surveillance delivered alongside lifestyle and psychological support by other team members, in person or remotely, is almost certainly the way forward. Further work is needed to understand whether emerging healthcare technologies (eg, wearables) improve or worsen patient experience. If ambulatory care can be provided in this way, it would focus the limited ACHD clinician resources to deliver diagnostic and treatment services to patients when their time of need comes.

## Data Availability

Data are available on reasonable request.
