# Supplementary material for: Outpatient care of adults with congenital heart disease in the UK: a qualitative appraisal of the clinician perspective
Source: Open Heart. 2024 Jan 29;11(1):e002420. doi: 10.1136/openhrt-2023-002420 (PMC10826567; doi:10.1136/openhrt-2023-002420)
Supplement: Supplementary data [file openhrt-2023-002420supp001.pdf]

Specialist Topic guide

Typical care methods:

How is your ACHD outpatient service organised?

For example, how often are clinics, how many patients come, what staff are involved.

How are patients advised to manage their condition outside of healthcare?

Describe a (general outpatient) clinic appointment with an adult congenital patient?

What do the different types of interaction with your patients look like?

How well do you think your ACHD outpatient system works?

Do you feel like this care works well for the patient?

How satisfied do patients seem with this level of care.

Do you feel like the resources are used appropriately? Are they wasted? Are they insufficient?

Would you change this interaction? How?

Can you summarise in a few sentences what you think are the key goals of outpatient care for adults with congenital heart disease?

Do you feel like you manage to achieve these with the current availability of resources?

If no, what is the limitation on resources? Who do you think is responsible?

A recent study (Coats & Chaudhry, 2021) found 80% of ACHD clinic appointments result in no decision other than continued surveillance. How does this compare to your personal experiences?

How do you feel about recurring clinic appointments without changing treatments?

What does a sufficiently cared for patient look like?

What would you need to do to achieve this level of care for your current patients?

Chaudhry I, *et al. Open Heart* 2024; 11:e002420. doi: 10.1136/openhrt-2023-002420

Describe the transition of patients from paediatric care to adult care at your centre. How does it compare with other centres that you know about.

How effective do you feel your transition process is?

What changes would you make if there were no financial limitations.

Are there any changes you would make that could save money or deliver better transition with the same cost?

Is nonattendance an issue for your clinic?

What percentage of appointments are missed?

What are the reasons that lead to nonattendance for your patients?

How do you manage non-attendance in your clinic?

Describe any changes that were made to care provision during Covid?

Do you plan to continue some or all, or will you return to previous systems?

Explain why.

Individuality of patient care:

What challenges do you face when caring for different adults with CHD?

Do you have to cater to different requirements based on culture?

How well do you manage these challenges?

Do you feel there would be a good way to plan for this?

Can you describe any differences in care provision for patients with differing complexity of congenital heart disease?

How well is care presently co-ordinated?

What aspects could be improved to meet individual needs?

How do you co-ordinate with cardiologist in other centres? Does this relationship work well?

Are there any difficulties you experience? What could improve things?

Is there anything special about the doctor-patient relationship for adults with CHD?

How do you manage patients who are not trusting of healthcare?

How do you manage patients who feel neglected by healthcare system?

What happens if a patient stops attending clinics?

Accessibility of services:

What do adults with congenital heart disease need to know about their condition?
Do you find any difficulty communicating particular aspects?
What are these, what could help you?
How is information made available to patients about their condition?
Is it provided to patients, or must they look for it?
How helpful do you think available resources are?
Do you feel like patients are educated sufficiently on how to care for themselves?

How frequently are patients encouraged to attend clinics?
What other types of care and services are available to your patients?
Do you feel like the current provision of outpatient care works?
Do patients need to travel for this - how far is a typical distance to travel?
Could care be provided in a different way?

Psychological Support:

Which aspects of a patient's condition/care do you believe cause them anxiety?
What causes anxiety and worry for you?

To what extent do you believe psychological support should be available to outpatients?
How are patients advised to support themselves in their condition?
How do you ensure patients are supporting themselves?
Do you provide psychological resources?
Is psychology support better provided in the specialist centre or primary care?

Describe how you would improve psychological care for patients?

Chaudhry I, *et al. Open Heart* 2024; 11:e002420. doi: 10.1136/openhrt-2023-002420

<u>Other Chronic Disease Models:</u>

What awareness do you have of how other adults with chronic disease manage their conditions, for example diabetes or cystic fibrosis?

Is this something that could work for adults with CHD?

What problems do you foresee for adults with CHD?

What aspects of healthcare could ACHD patients self-manage?

Which areas should stay under clinician control?

Could anyone else help support adults with CHD in managing their health?

Which mobile health technologies might be suitable for use in adults with CHD?

How does primary care presently support adults with CHD?

What are the problems with how this system works at present?

What could work better?

Could a more proactive role be developed?

Is there anything we haven't covered which might be important to think about in providing ambulatory care for adults with CHD?

Non-specialist Cardiologist topic guide:

<u>Typical care methods:</u>

Can you describe your experience of interacting with adult congenital heart disease patients?

How frequently do you care for ACHD patients, what does this look like?

How is care for these patients organised in your hospital?

How does your interaction with the specialist centre work?

What conditions do you feel happy managing in your centre? What do you need help with?

Chaudhry I, *et al. Open Heart* 2024; 11:e002420. doi: 10.1136/openhrt-2023-002420

What are you uncomfortable managing?
How common do you believe ACHD to be? – Incidence of 3-4/1000.

What are your goals when interacting with ACHD patients?
Are resources easily accessible to support you in your care for them?
Can you get the advice you need? Have you ever experienced problems knowing how to get the right advice?

How do you feel about patient care in hospital outpatient clinics?
How does the current outpatient clinic care provision affect the care you provide?
Do you feel like patient experience could be improved through a change in your role? How?

A recent study (Coats & Chaudhry) suggested 80% of hospital appointments for ACHD patients result in no decision other than continued surveillance.
What are your thoughts about this? How do hospital clinics affect your workload?
Other than outpatients, in what other ways might surveillance work well?

What do you feel are the costs to patients with the current way specialist and general cardiology outpatients and primary care function? What are the costs for healthcare providers? Is there a different way to do things that might work better?

What does a sufficiently cared for patient look like?
How do you achieve this level of care for a patient?
How do you feel about the current number of patients receiving insufficient care?

Do you feel like patients are satisfied with their level of care?
Do you feel like the current care system is sufficient for patients?
What resources are provided to patients? Are they used sufficiently?

Are there changes during COVID that could be continued that you have seen resulting in improved satisfaction and reduced burden for patients or healthcare providers?

Individuality of patient care:

What challenges do you face when caring for different adults (with or without CHD)? May need to provide examples (ethnicity, learning difficulties, mental health, socioeconomic status)

Do you have to cater to different requirements based on culture etc.?
Do you have support systems in place that help with this?
How do you manage these challenges?

How well is care presently co-ordinated between centres and what aspects could be improved to meet individual patient needs?

Accessibility of services:

What challenges do you face when caring for different adults in your clinic?
Do you have to cater to different requirements based on culture?
How well do you manage these challenges?
Do you feel there would be a good way to plan for this?

How well informed about their condition are adults with congenital heart disease in your experience?

Do you notice that some people with certain conditions are better informed than others? Can you describe some of these differences?
Do you ever have difficulty communicating particular aspects of congenital heart disease? Can you give an example? What type of information do you find works well for informing patients?
Do you feel like patients are educated sufficiently on how to care for themselves?

Psychological Support:

What aspects of a patient's condition/care do you believe cause them anxiety?
What causes anxiety and worry for you?

Chaudhry I, *et al. Open Heart* 2024; 11:e002420. doi: 10.1136/openhrt-2023-002420

To what extent do you believe psychological support should be provided to those with chronic disease? What is your experience of anxiety and depression in those with chronic conditions?

How are patients advised to support themselves in their condition? How do you ensure patients are supporting themselves?

Do you provide psychological resources? Where should they best be provided?

Describe how you would improve psychological care for patients?

Other Chronic Disease Models:

What is your awareness of how other adults with chronic disease manage their conditions, for example diabetes or cystic fibrosis?

Is this something that could work for adults with CHD? What problems do you foresee for adults with CHD?

What do you think is the present role for primary care in supporting adults with CHD?

What are the problems with how this system works at present?

What could work better? Could a more proactive role be developed?

What aspects of healthcare could ACHD patients self-manage?

Do you have experience of other cardiac patients who self-manage aspects of their condition? What things work well? Which areas should stay under clinician control?

Could anyone else help support adults with CHD in managing their health?

Do you have experience of using any mobile health technologies in adults with cardiac disease?

What has worked well? What has caused problems? Do patients like mobile technology?

Is there anything we haven't covered which might be important to think about in providing ambulatory care for adults with CHD?

GP prompt guide

<u>Typical care methods:</u>

Could you describe any experience you have of patients with adult congenital heart disease? (Or chronic illnesses/heart conditions if not)

- What does your interaction with patients look like?
- How much responsibility do you FEEL you have for patients with a heart condition/chronic condition, as a primary care provider?
- Could you describe any changes you would make to the current level of interaction you have with patients with heart conditions/chronic conditions? How?

How common do you think ACHD is? - Incidence of 8/1000 people in developed countries.

- Do you feel like you are aware of all the patients who are registered with your practice who have ACHD?
- Is there a plan for managing common issues in ACHD ie pregnancy, birth control, exercise? Do you feel like a plan would improve care?

Could you describe how you presently support adults with CHD in the primary care service?

- What are the problems with how this system works at present?
- What could work better?
- Could a more proactive role be developed?

What are your key goals when interacting with patients in general?
Does this change at all when interacting with patients with heart conditions?

- Do you feel like you manage to achieve these goals with the current availability of resources?

What does a sufficiently cared for patient look like? Is this different for a patient with a known heart condition/chronic condition?

- How do you achieve this level of care for a patient?

Chaudhry I, *et al. Open Heart* 2024; 11:e002420. doi: 10.1136/openhrt-2023-002420

Could you describe the changes that were made to maintain patient care during COVID?

- Could any of these changes be continued post-pandemic?

<u>Individuality of patient care:</u>

What challenges do you face when caring for different patients?

- Do you have to cater to different requirements based on culture, age etc? do you feel there would be a good way to plan for this?
- Are there challenges due to these different requirements?
- How do you manage these challenges?
- How relevant is a patients chronic/heart condition when they approach you for general queries/health advice?
- Do you approach patients with chronic conditions (heart conditions) differently?
- Is the level of a patient’s education on their condition a factor in this?

To what extent do you incorporate patient’s own knowledge of their condition into care?

Would you change this?

How well is care presently co-ordinated and what aspects could be improved to meet individual needs?

Do you feel you are provided with sufficient information regarding ACHD/heart condition patient’s condition, in order to properly care for them?

Do you feel you have sufficient contact/communication with other areas/practitioners of the healthcare service in order to properly care for patients?

Would you change the co-ordination of care in any way?

<u>Psychological Support:</u>

Which aspects of a patient (with a chronic condition) condition/care do you believe cause them anxiety?

Chaudhry I, *et al. Open Heart* 2024; 11:e002420. doi: 10.1136/openhrt-2023-002420

BMJ Publishing Group Limited (BMJ) disclaims all liability and responsibility arising from any reliance placed on this supplemental material which has been supplied by the author(s)

What causes anxiety and worry for you?

To what extent do you believe psychological support should be available to outpatients?

- How are patients advised to support themselves in their condition?
- How do you ensure patients are supporting themselves?
- Do you provide psychological resources?

Describe how you would improve psychological care for patients with chronic illnesses?

<u>Other Chronic Disease Models:</u>

Can you describe any awareness you have of how other adults with chronic disease manage their conditions, for example diabetes or cystic fibrosis?

- Is this something that could work for adults with CHD?
- What problems do you foresee for adults with CHD?

What is your opinion of patients with chronic illnesses self-managing their condition?

- Which areas should stay under clinician control?

Which emerging mobile health technologies might be suitable for use in adults with CHD?

Is there anything we haven't discussed you believe to be important when thinking about ACHD outpatient care?

Chaudhry I, *et al. Open Heart* 2024; 11:e002420. doi: 10.1136/openhrt-2023-002420
